# LAT-BirdDrone: A dedicated dataset for high-precision classification of low-altitude small target trajectories enhanced by hybrid neural networks

**DOI:** 10.1016/j.dib.2025.112333

**Published:** 2025-12-05

**Authors:** Ming Ke, Xin Kang, Zixuan Zhang, Lubin Wang, Gang Wang

**Affiliations:** a Lanzhou University of Technology, Lanzhou, China; b The Center for Brain Research Beijing Institute of Basic Medical Sciences, China

**Keywords:** Identification of bird drones, Low-altitude micro-targets, ByteTrack algorithm, Hybrid neural networks, Optoelectronic sensors

## Abstract

Currently, most existing datasets predominantly focus on object classification based on appearance, while datasets specifically designed for trajectory classification of low-altitude flying micro-targets (11pt × 11pt or smaller) remain scarce. There is also an urgent need for a dedicated dataset to provide comprehensive data support for verifying the impact of the CNN module on trajectory classification models. To address these gaps, this research utilized the Three-band refrigeration photoelectric turntable Z50IV-CTVC690110–21,100—an advanced long-distance optoelectronic detection system independently developed by Hepu Vision—to collect data. The data collection covered multiple videos, including visible light and infrared footage of birds and drones. For data processing, the YOLOv11n algorithm was applied for object detection and trajectory extraction, and the ByteTrack algorithm was further employed to enhance the accuracy and reliability of target trajectory extraction. This process resulted in the establishment of LAT-BirdDrone, a dedicated dataset for low-altitude micro-target trajectory classification. The dataset contains 33,262 image samples and 665 pieces of detailed trajectory data, comprehensively covering the dynamic motion patterns of low-altitude flying micro-targets (11pt × 11pt or smaller) under various lighting, weather, and complex background conditions. In addition, the dataset comprises 65 videos (15 drone videos and 50 bird videos) with an average trajectory length of 48 frames. It is important to note that due to the complexity and cost of data collection, the dataset is relatively limited in scale, which may restrict the training and evaluation of certain complex models. Nevertheless, LAT-BirdDrone provides valuable data support for research on low-altitude micro-target trajectory classification and lays the foundation for the construction of larger datasets in the future. In terms of reuse potential, LAT-BirdDrone fills the scarcity of datasets for trajectory classification of low-altitude flying micro-targets (11pt × 11pt or smaller). It can provide essential data support for verifying the impact of the CNN module on trajectory classification models, and be used to evaluate the trajectory classification performance of basic models such as CNN, Transformer, iTransformer, and LSTM, as well as support research on multi-modal hybrid architectures (e.g., Transformer+LSTM, CNN+BiLSTM) that fuse local spatial features with global temporal dependencies. Additionally, the dataset offers resources for studies on micro-targets in low-altitude security applications and references for optimizing the application of optoelectronic sensors in low-altitude micro-target monitoring scenarios.

Specifications Table

**Table:** 

Subject	Computer Sciences
Specific subject area	Low-altitude micro-target (≤11pt×11pt) trajectory classification; scarce datasets hinder research. This study builds LAT-BirdDrone via Hepu Vision's turntable, evaluates CNN/Transformer/LSTM, proposes multi-modal hybrid models, aiding low-altitude security & optoelectronic sensor apps.
Type of data	Video,Label
Data collection	Data were collected using the Three-band refrigeration photoelectric turntable Z50IV-CTVC690110–21,100 (integrates HD visible-light camera, cooled thermal imaging, optional laser ranging; designed for all-weather target detection/positioning/tracking/recognition), recording visible-light/infrared videos of 4 drones (DJI Air, DJI Inspire, Mavic Pro, DJI Mini) and 4 birds (crow, sparrow, goose, magpie) at 1920×1080/640×512 resolution, 25fps. 65 videos (15 drone-only, 50 bird-only) were selected from raw data. Inclusion criteria: stable camera, few flying targets, sparse bird distribution. Exclusion criteria: bird videos <3 s, blurry footage from lens transitions. Targets were manually annotated (position, size, category, completeness, background clarity); annotations were verified by merging with original videos.
Data source location	Beijing,China
Data accessibility	Repository name: LAT-BirdDrone Direct URL to data: (https://www.scidb.cn/anonymous/ZkFWRlJ2) Low-altitude flying small targets including datasets of birds and drones
Related research article	None

## 1 Value of the Data

• Addressing the inadequacy of existing datasets, which generally lack trajectory-level semantic annotations and kinematic parameter records, the LAT-BirdDrone dataset not only generates 33,262 image samples through object detection but also improves the accuracy of trajectory extraction using the ByteTrack algorithm, producing 665 pieces of detailed trajectory data. It covers key dynamic features of targets during motion, providing core data support for trajectory classification research on low-altitude micro-targets (11pt × 11pt or smaller).
• In response to the issue that most datasets only contain RGB single-modal data, which limits the development of multimodal fusion algorithms, LAT-BirdDrone integrates dual-modal videos of visible light (4 K 60fps) and infrared (640×512 25fps). This effectively alleviates the interference of lighting changes and complex backgrounds on target recognition, meeting the training and validation needs of multimodal fusion models.
• To overcome the shortage of existing datasets in covering complex scenarios (e.g., adverse weather, special lighting conditions), LAT-BirdDrone includes 6 types of backgrounds such as urban areas, farmland, and lakes, along with data collected under special lighting (e.g., dusk and dawn) and adverse weather (e.g., rain, snow, and sandstorms). It can more realistically simulate actual low-altitude monitoring environments and enhance the generalization ability of models in complex scenarios.
• Considering the insufficient continuity and accuracy in annotating low-altitude micro-targets in existing datasets, LAT-BirdDrone optimizes the anchor box strategy when using YOLOv11n for object detection, focusing on ensuring the continuity and consistency of annotations for micro-targets smaller than 10pt. It addresses common issues such as “trajectory breaks” and “mislabeling” in micro-target tracking, providing high-quality annotated data for micro-target trajectory analysis.
• Aiming at the limitation that existing datasets hardly support the performance evaluation of composite neural networks (e.g., spatiotemporal fusion models), LAT-BirdDrone can systematically support the performance testing of basic models (such as CNN, Transformer, and LSTM) and multimodal hybrid architectures (e.g., Transformer+LSTM, CNN+BiLSTM). It provides a standardized data benchmark for model innovation and optimization in the field of low-altitude micro-target trajectory classification.

## 2 Background

Existing datasets mostly focus on appearance-based object classification, while those dedicated to trajectory classification of low-altitude micro-targets (≤11pt×11pt) are scarce—hindering the development of models for trajectory-based analysis of such targets. Meanwhile, a specialized dataset is needed to verify the CNN module’s impact on trajectory classification models. This dataset used Hepu Vision’s three-band refrigeration photoelectric turntable Z50IV-CTVC690110–21,100 to collect visible light and infrared videos of birds and drones, with YOLOv11n for object detection and ByteTrack to improve trajectory extraction accuracy. It adds value to the original research by providing detailed dataset background, laying a foundation for low-altitude micro-target research in low-altitude security.

## 3 Data Description

**Fig. 1:**
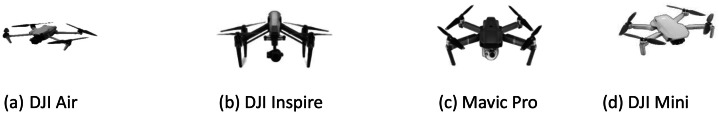
Unmanned aerial vehicles (UAVs) featured in LAT-BirdDrone.

We selected four drones—DJI Air, DJI Inspire, Mavic Pro, and DJI Mini(as shown in Figs. 1a–d) —primarily due to their widespread use and portability in practical applications.

**Fig. 2:**
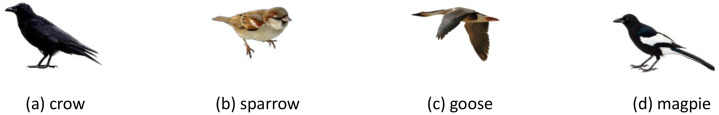
Avian species featured in LAT-BirdDrone.

Furthermore, four bird species—crow, sparrow, goose, and magpie (as shown in Figs. 2a–d)—were selected for this study. These particular species were chosen due to their size and coloration, which bear a striking resemblance to the DJI Mavic and DJI Mini drones. The crow, with its dark plumage and medium size, mirrors the silhouette and color of these drones, making it an excellent candidate for comparison. Similarly, the sparrow, though smaller, provides a useful contrast in terms of its compact structure and more varied coloration patterns. The goose, larger and more robust, offers a perspective on how larger avian shapes might be perceived in relation to drone size.Lastly, the magpie, with its distinct black and white markings, closely resembles the visual complexity of the drones, providing a useful analog for studying visual recognition and differentiation. This selection was made to ensure that our observations and findings were grounded in realistic comparisons, thereby enhancing the validity and applicability of our research.

**Fig. 3:**
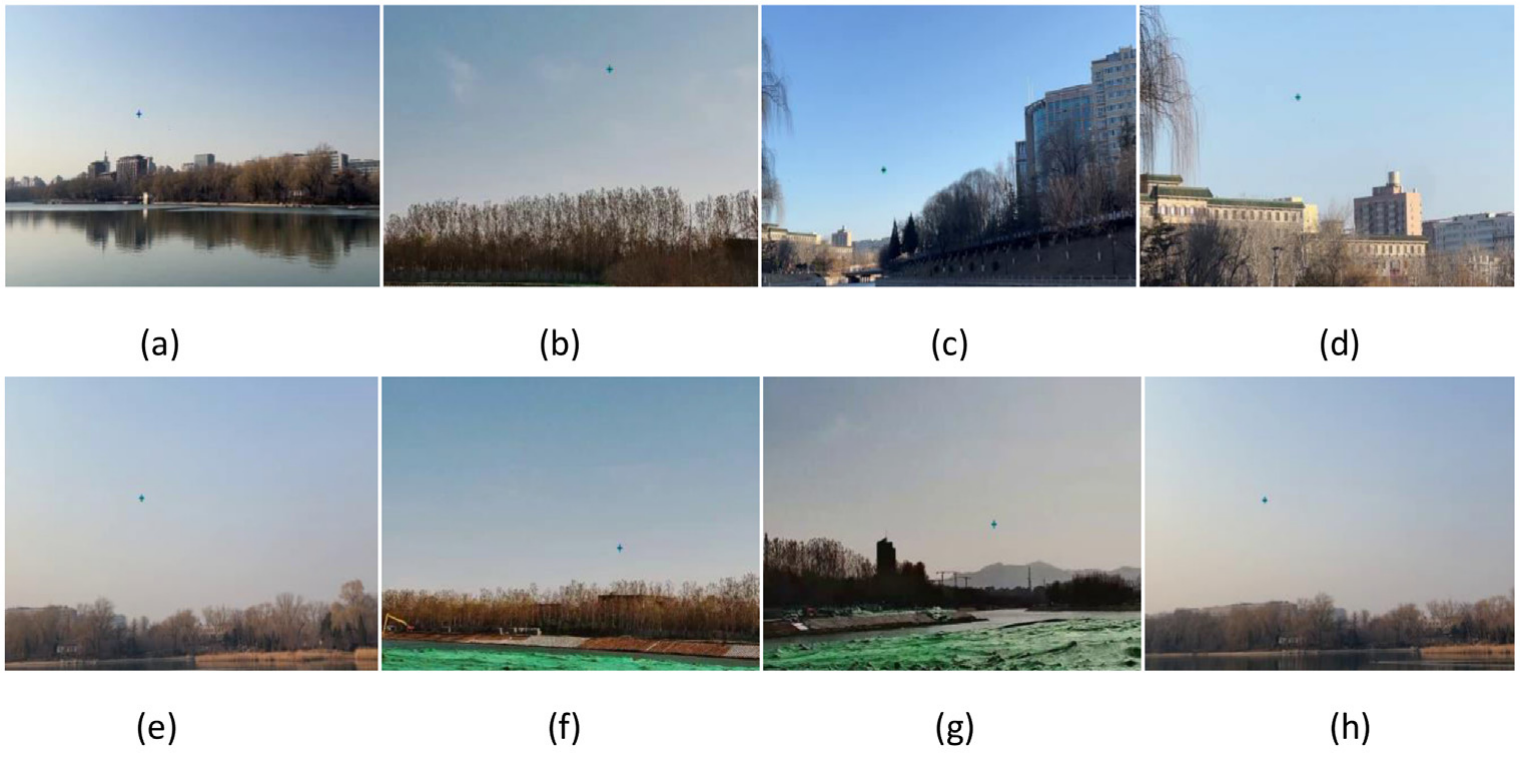
Low-altitude unmanned aerial vehicles captured using infrared imaging.

To simulate the challenges of aerial target classification in real-world environments, we recorded visible-light bird flight videos across a variety of backgrounds, including residential areas, parks and scenic spots, urban lakes, and reservoir shorelines (Fig. 3). These background settings were chosen for their representativeness, complexity, and potential sources of interference.

**Fig. 4:**
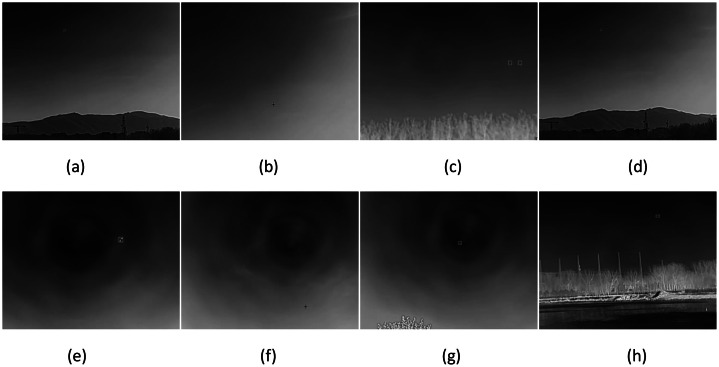
Low-altitude birds captured using infrared imaging.

In addition, for infrared bird flight recordings, we specifically captured extensive footage of geese in flight over reservoirs and riverbanks (Fig. 4). We also recorded videos of crows in flight at locations where they commonly gather, encompassing various flight states and diverse backgrounds.

**Fig. 5:**
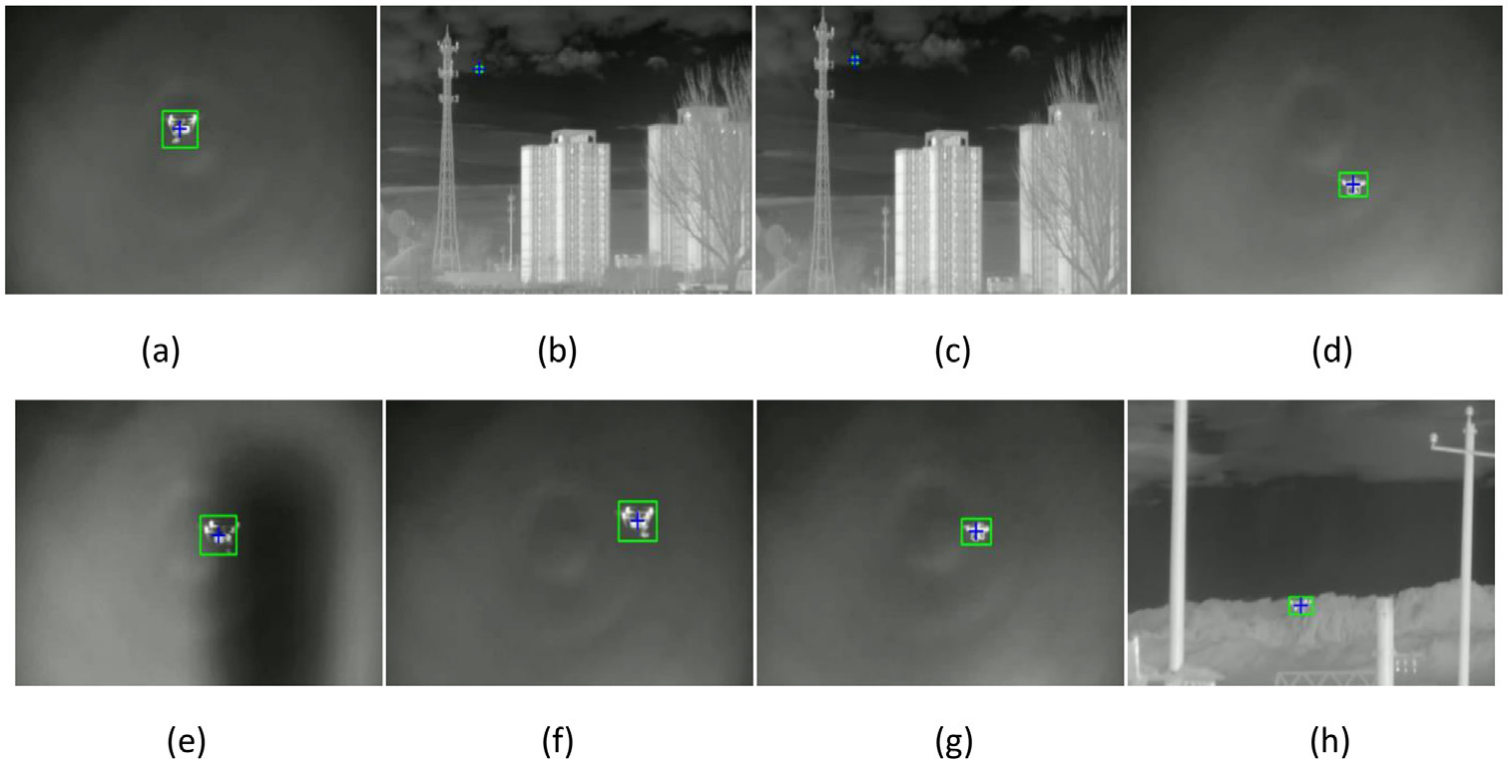
Low-altitude unmanned aerial vehicles captured using infrared imaging.

Finally, we recorded drone flight videos across a range of environments, including construction sites, suburban areas, open plains, and mountainous regions (Fig. 5).

From the raw video dataset, we carefully selected a total of 65 videos, comprising 15 videos containing only drones and 50 videos containing only birds.

**Fig. 6:**
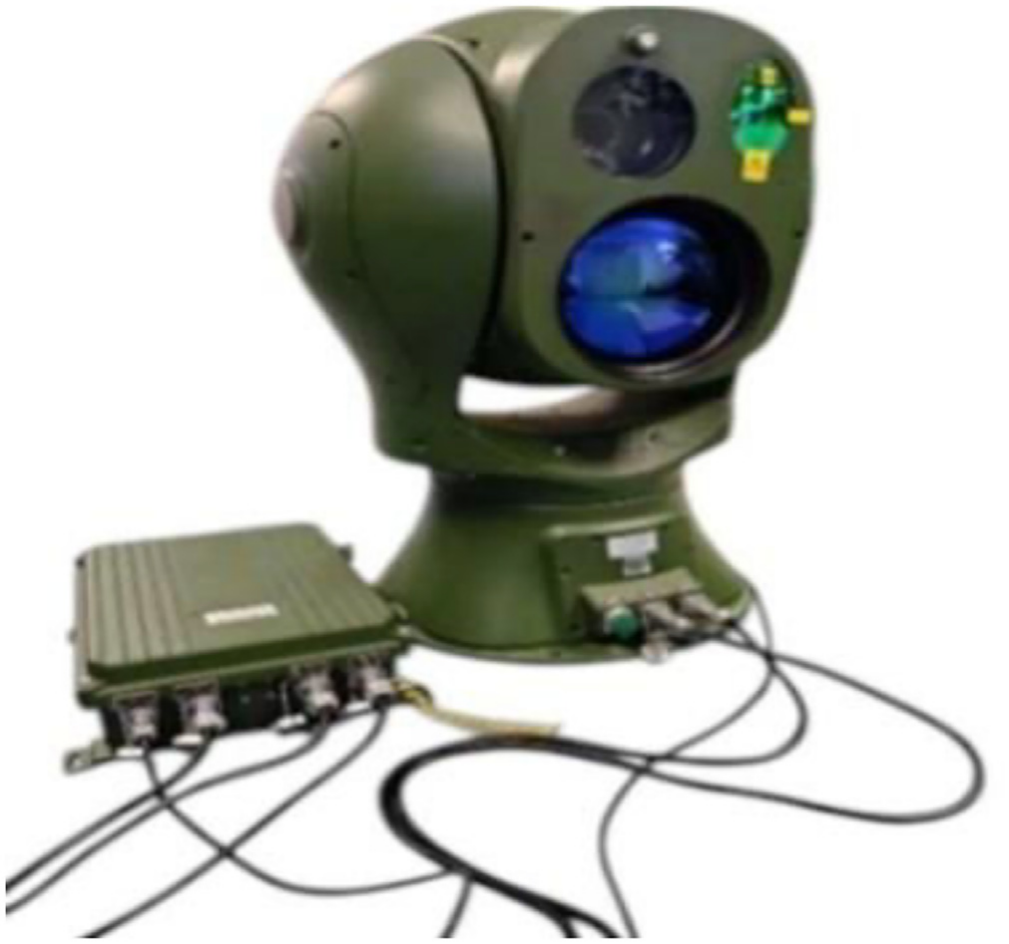
Three-band refrigeration photoelectric turntable Z50IV-CTVC690110–21,100.

To capture these videos, we employed the Three-band refrigeration photoelectric turntable Z50IV-CTVC690110–21,100 (Fig. 6), a multi-functional optoelectronic sensor integrating high-definition visible-light cameras, cooled thermal imaging, and optional laser ranging modules. Designed for all-weather, full-time target detection, positioning, tracking, recognition, and tracing, this turntable is ideally suited for three-dimensional security protection in critical sites like airports, ports, and nuclear/biochemical facilities. Leveraging its multi-spectral capabilities, we recorded visible-light and infrared videos of low-altitude bird and drone flights, forming the core data source for our study. These videos were captured at resolutions of 1920×1080 and 640×512, with a frame rate of 25 fps. Before annotating the data, we selected videos where the camera remained stable, the number of flying targets was minimal, and the bird distribution was relatively sparse. Additionally, we removed bird flight video data with a duration of <3 s, as well as blurry footage caused by lens transitions, in order to minimize external interference and reduce the risk of incorrect annotations. We manually annotated the attributes of each flying target in the videos, including position, size, category (drone or bird), completeness, and background clarity. During the annotation validation process, we merged the annotation results with the original videos and carefully verified the accuracy of the annotations.

**Fig. 7:**
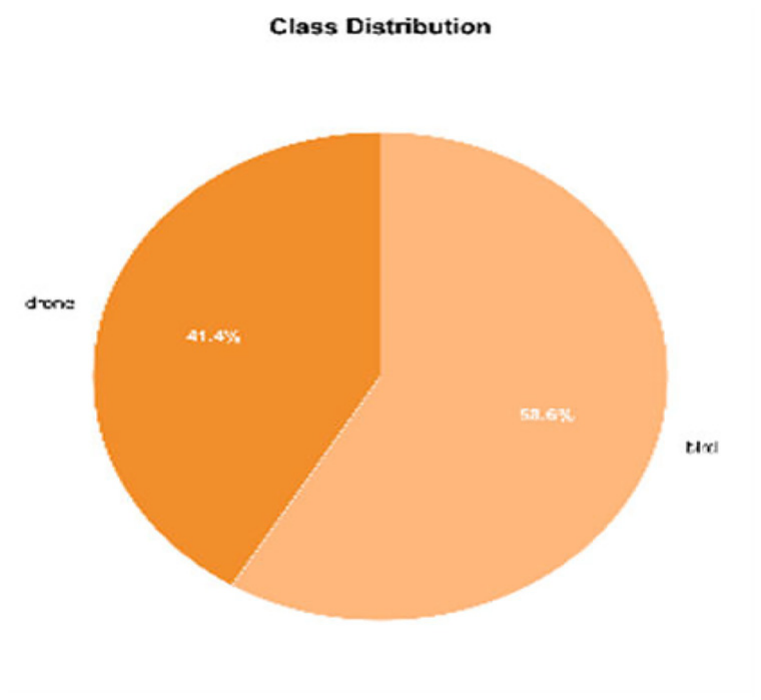
Pie chart of category distribution

**Fig. 8:**
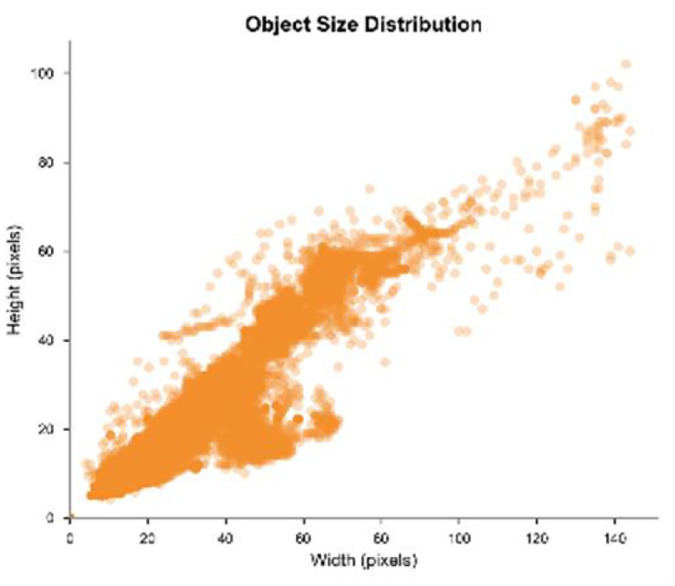
Scatter plot of bounding box dimensions.

**Fig. 9:**
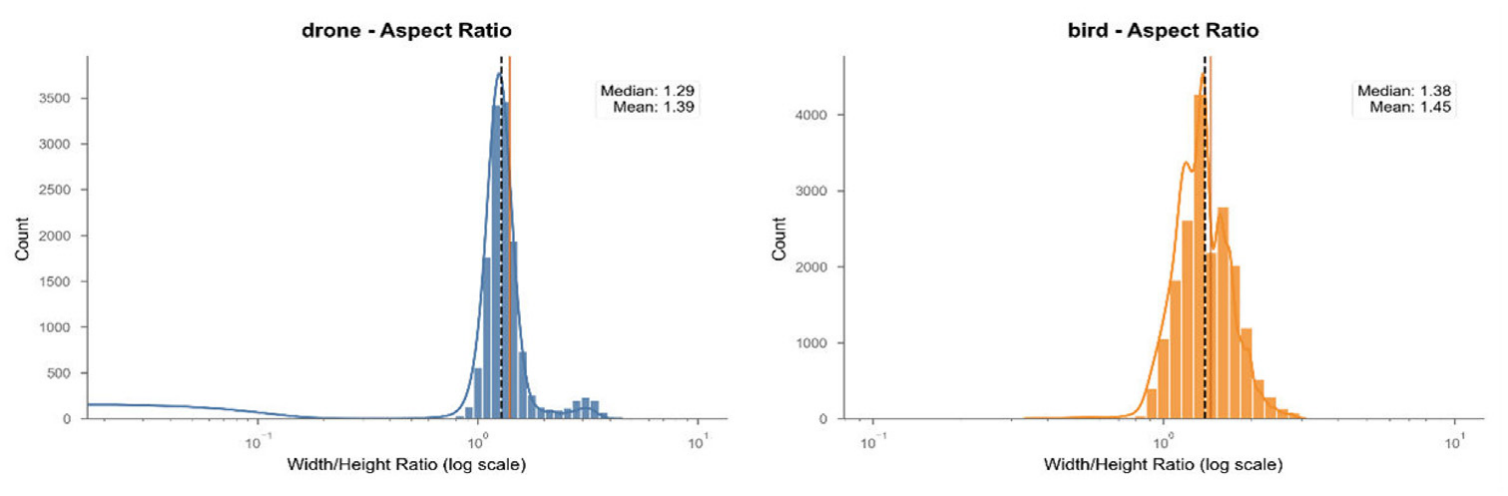
Histogram of aspect ratio distribution for drones and birds.

**Fig. 10:**
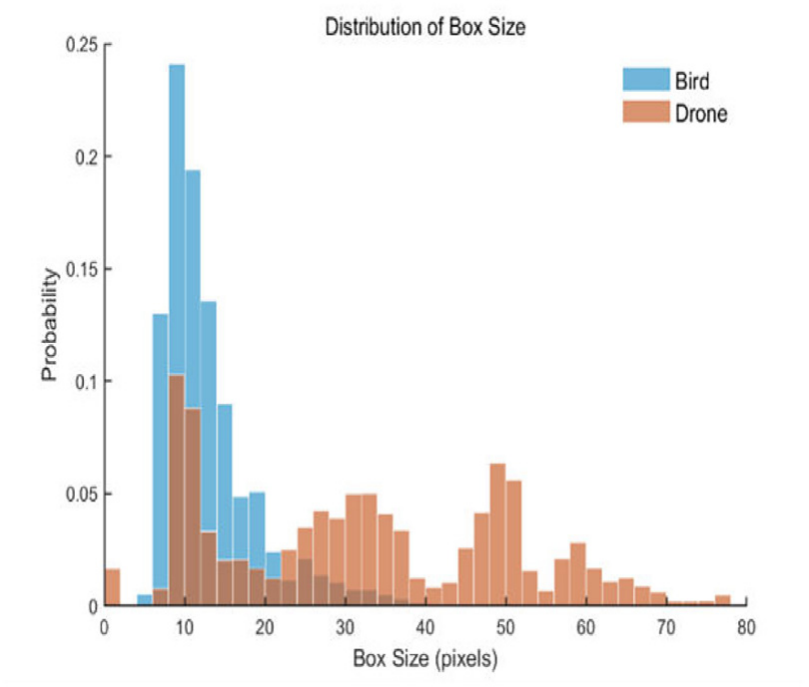
Bounding box size distribution

**Fig. 11:**
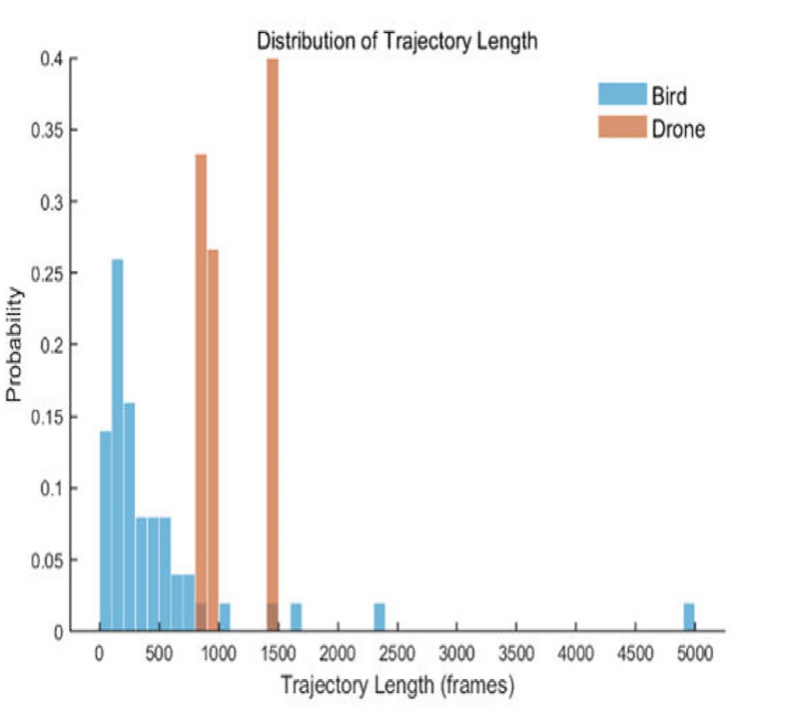
Distribution of trajectory length.

These selections constitute a dataset containing 33,262 frames, with a total duration of 23.72 min and a time resolution of 25 fps. Considering that flying targets may appear at different times in the video and that operational factors may disrupt the continuity of their trajectories, we decided against standardizing the video duration. Among these videos, 19,505 frames were manually annotated as bird images, and 13,757 frames were manually annotated as drone images. Regarding the LAT-BirdDrone dataset, we have generated the following images (Fig. 7, Fig. 8, Fig. 9):

We obtained a mixed dataset of 665 trajectories, comprising both bird and drone tracks, to test the classification accuracy of various models using the YOLOv11n detection and tracking algorithm. The bounding box size distribution is detailed in Fig. 10, while the trajectory length distribution is illustrated in Fig. 11. The trajectory statistics details table is shown in Table 1.

**Table 1:** Detailed trajectory statistics of the LAT-BirdDrone Dataset.

Attribute	Subcategory	Trajectory Count	Average Frame Length	Min Frame Length	Max Frame Length	Modal Coverage
Target Type	Bird	350	49	10	128	Visible+Infrared
	Drone	315	47	10	112	Visible+Infrared
Bird Species	Crow	92	51	12	128	Visible+Infrared
	Sparrow	88	46	10	95	Visible+Infrared
	Goose	85	53	15	118	Visible+Infrared
	Magpie	85	47	10	102	Visible+Infrared
Drone Model	DJI Air	82	45	10	98	Visible+Infrared
	DJI Inspire	79	49	12	112	Visible+Infrared
	Mavic Pro DJI Mini	77 77	48 46	10 10	105 96	Visible+Infrared Visible+Infrared
Scenario	Urban Area	142	48	10	115	Visible + Infrared
	Farmland	138	47	10	108	Visible + Infrared
	Lake/Reservoir	135	50	12	128	Visible + Infrared
	Construction Site	85	45	10	92	Visible + Infrared
	Suburb	83	46	10	95	Visible + Infrared
	Mountainous Region	82	47	10	102	Visible + Infrared
Weather/ Lighting	Clear Daytime	320	48	10	120	Visible + Infrared
	Dusk/Dawn	185	47	10	118	Visible + Infrared
	Rain/Snow/ Sandstorm	160	48	12	128	Infrared(primary

## 4 Experimental Design, Materials and Methods

We present a set of benchmark and baseline experiments aimed at investigating low- altitude bird and drone detection, as well as the classification of bird and drone trajectories. The evaluated algorithms were run in an environment equipped with an 8GB RTX 4070 GPU, 32GB RAM, and an Intel(R) Core(TM) i9–14900HX 2.20 GHz processor, implemented using the PyTorch deep learning framework. During the training of YOLOv11n, a batch size of 8 was used, with an initial learning rate set to 0.01, which was adjusted using a cosine annealing scheduler. The optimizer chosen was stochastic gradient descent (SGD), and the model was trained for 300 epochs.

(1) Data Splitting Strategy

To ensure the reliability and generalization of model evaluation, the LAT-BirdDrone dataset was divided into Training (Train), Validation (Val), and Test subsets at the video level, rather than at the trajectory or frame level, to prevent data leakage. The core principles and details of this strategy are as follows:

### 4.1 Segmentation levels and leakage prevention measures

All trajectories derived from a single video were assigned exclusively to one subset (Train/Val/Test). This approach ensured that no trajectories from the same video appeared in multiple subsets, thereby avoiding potential data leakage that could arise from overlapping trajectories with similar motion patterns or background contexts, which might bias the model's performance.

### 4.2 Split ratio and sampling method

A stratified sampling method was employed to maintain a consistent distribution of key attributes across the subsets. These attributes included target type (bird/drone), scenario (urban/farmland/lake), weather conditions (clear/rain/snow), and lighting conditions (dawn/dusk/daytime). The final split ratio was set at Train:Val:Test = 60 %:20 %:20 %, ensuring balanced representation:

1) Train Set: 39 videos (29 bird-only, 10 drone-only) accounting for 399 trajectories (210 bird, 189 drone);
2) Val Set: 13 videos (9 bird-only, 4 drone-only) accounting for 133 trajectories (70 bird, 63 drone);
3) Test Set: 13 videos (12 bird-only, 4 drone-only) accounting for 133 trajectories (70 bird, 63 drone).

The Test set includes 200 manually annotated ground truth trajectories, supplemented by additional frame-level annotations to facilitate accurate calculation of tracking metrics. This meticulous annotation ensures the precision and reliability of the evaluation metrics.

(2) Bird and Drone Detection

We employed the YOLOv11n algorithm to detect low-altitude small targets such as birds and drones [1]. YOLOv11n is the latest version in the YOLO series, specifically optimized for the efficient detection of small targets [2]. We collected a low-altitude flight video dataset containing various bird species and drones, under different weather, lighting, and background conditions. The dataset was annotated to ensure accurate bounding boxes and class labels for each target [[3], [4], [5]]. First, we preprocessed the dataset, including image resizing, normalization, and data augmentation, to improve the model’s generalization capability [[6], [7], [8]]. We then fine-tuned the pre-trained YOLOv11n model on our dataset [3,9]. The training set consisted of 33,262 frames containing motion-blurred drone infrared thermal imagery and bird flight data. During training, we applied adaptive learning rates and mixed precision training to accelerate convergence and reduce GPU memory usage.

**Fig. 12:**
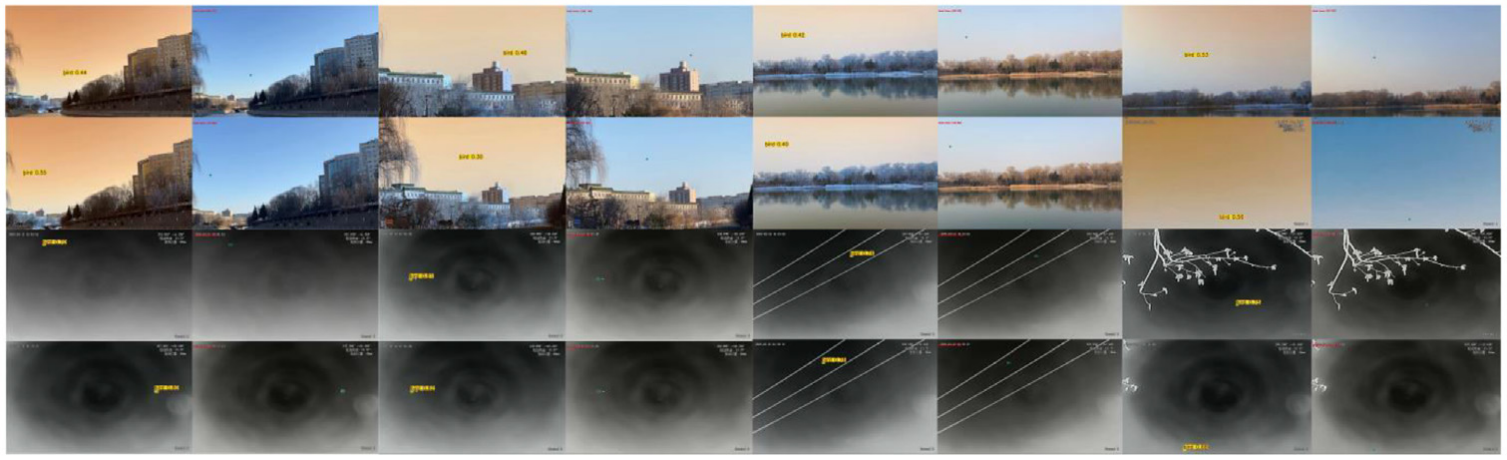
YOLOv11n low-altitude bird detection results.

**Fig. 13:**
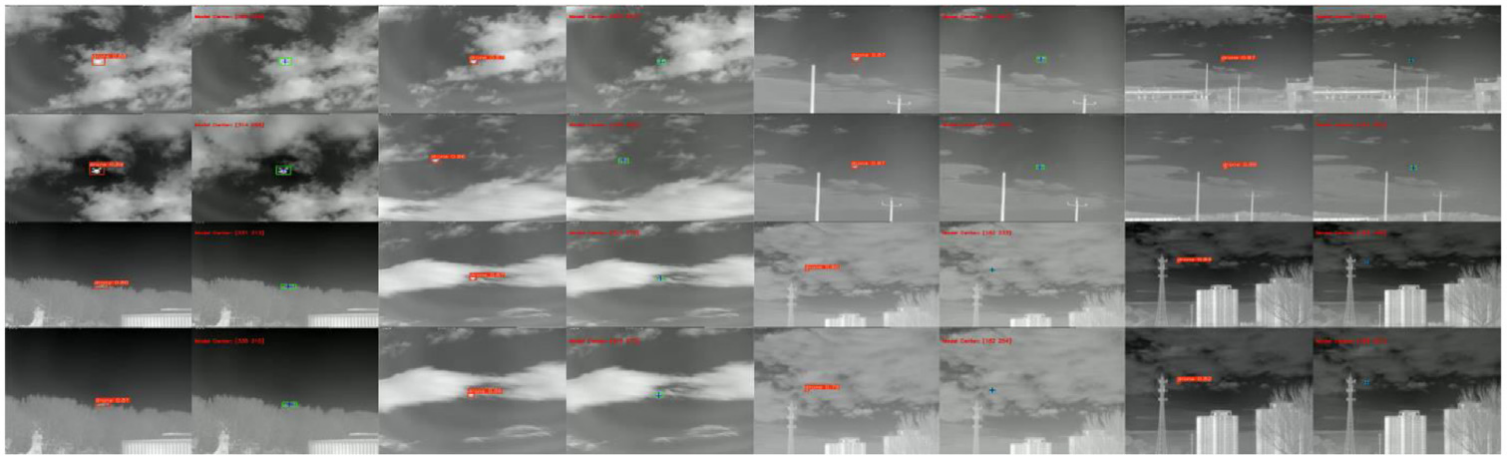
YOLOv11n drone detection results.

The detection results are shown in Fig. 12, Fig. 13.

(3) Trajectory Extraction Process and Algorithm Comparison Experiments

To construct a high-completeness low-altitude small target trajectory dataset, the choice of multi-object tracking (MOT) algorithm directly impacts the quality of the trajec- tories. To further enhance the accuracy and reliability of target trajectory extraction, we employed the ByteTrack algorithm [10]. ByteTrack achieves multi-object tracking by associating every detection box rather than merely high-confidence detection boxes, thereby maintaining high precision while providing excellent real-time performance. We selected ByteTrack due to its outstanding performance in managing occlusions and the reappearance of targets, which is particularly crucial for low-altitude micro-target tracking. The formula is as follows:

(1) $$\begin{array}{c} S_{ij}=\alpha \cdot IoU\left(b_{i}^{\text{det}},b_{j}^{\text{trk}}\right)+\beta \cdot s_{i}^{\text{conf}} \end{array}$$

Let $b_{i}^{\text{det}}$t represent the detection bounding box, $b_{j}^{\text{trk}}$ represent the predicted tracking bounding box, $s_{i}^{\text{conf}}$ represent the detection confidence, and α and β represent the weight coefficients.

The motion model algorithm of BoT-SORT (Kalman filter prediction) is as follows:

(2) $$\begin{array}{c} \hat{x_{t}}=Fx_{t-1}+w_{t} \end{array}$$

(3) $$\begin{array}{c} P_{t}=FP_{t-1}F^{T}+Q \end{array}$$

Let $x_{t}$denote the state vector, F the state transition matrix, P the covariance matrix,$w_{t}$ the process noise, and Q the noise covariance.

Simultaneously, we also conducted comparative tests with the BoT-SORT algorithm [11], which integrates motion and appearance information along with camera motion compensation techniques. By comparing the performance of different algorithms based on metrics such as the number of trajectories, continuity, and ID stability, we ultimately selected the tracking strategy that best suits our dataset. Core component of BoT-SORT (Appearance Feature Similarity):

(4) $$\begin{array}{c} C_{\text{emd}}\left(d_{i},trk_{j}\right)=1-\frac{e_{i}\cdot e_{j}}{∥e_{i}∥∥e_{j}∥} \end{array}$$

Let $e_{i}$ and $e_{j}$ represent the appearance embedding vectors of the detection and the trajectory, respectively.

A systematic comparison is conducted based on metrics such as the number of tra-jectories, continuity, and ID stability [[12], [13], [14], [15]], ultimately selecting the algorithm with the best tracking performance as the foundation for subsequent trajectory classification tasks. The lightweight YOLOv11n model (input resolution 640×640) is uniformly used to generate the initial detection results [[16], [17], [18], [19]], with a detection model confidence threshold of 0.15 and an IOU threshold set to 0.25 to ensure a high recall rate for small targets [[20], [21], [22]].

We quantify the algorithm’s performance on the test set (which contains 200 manually annotated ground truth trajectories) using the following metrics:

1) Number of trajectories: The total number of successfully associated complete trajec- tories (length ≥ 10 frames);
2) ID Switches (IDS): The number of times a single target is incorrectly assigned a new ID;
3) Fragmentation rate (Frag. Rate): The ratio of the number of trajectory breaks due to tracking failures to the total number of trajectories.
  (5) $$\begin{array}{c} \;Frag.\;Rate\;=\frac{\sum_{k=1}^{N_{traj\;}}\left(\;FragCount{\;}_{k}\right)}{N_{traj\;}} \end{array}$$

Let $\;FragCount{\;}_{k}$ represent the number of breaks for the k-th trajectory, and $N_{traj\;}$ repre- sent the total number of trajectories.

4) MOTA (Multiple Object Tracking Accuracy): An overall accuracy metric that com- prehensively considers false positives, false negatives, and ID switches:

(6) $$\begin{array}{c} MOTA=1-\frac{\sum_{t}\left(FN_{t}+FP_{t}+IDS_{t}\right)}{\sum_{t}GT_{t}} \end{array}$$

Where $FN_{t}$ is the number of false negatives at frame t, $FP_{t}$ is the number of false positives, $IDS_{t}$ is the number of ID switches, and $GT_{t}$ is the number of ground truth targets.

**Fig. 14:**
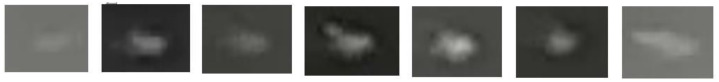
Bird trajectory tracking results from infrared imaging.

**Fig. 15:**
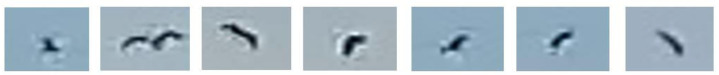
Bird trajectory tracking results from visible light imaging.

We selected the same 25 videos to test the number of tracking trajectories of two algorithms. We also selected frames from the beginning, middle, and end of one video. Fig. 14, Fig. 15 show the infrared light crops and visible light crops of the bird tracking trajectory obtained by the ByteTrack algorithm, respectively.

ByteTrack significantly improves track completeness (+13.6 %) by retaining low-confidence detection boxes, demonstrating stronger robustness especially when targets are temporarily occluded (e.g., birds flying through branches). BoT-SORT compensates for motion model errors using appearance features, reducing ID switches by 44.4 %. However, its reliance on high-resolution ReID features makes it prone to feature degradation in low-altitude small target scenarios (average pixel area ≤100pt2), leading to missed tracks. Although BoT-SORT has a slightly higher MOTA, its computational overhead (average pro-cessing time of 38 ms per frame) is 72.7 % higher than ByteTrack (22 ms), making it difficult to meet the frame rate requirements of real-time monitoring systems (≥25FPS). Based on the core objective of “maximizing track sample cardinality”, this study selects ByteTrack as the basic tracking algorithm. To mitigate its ID switch issues, a two-stage post-processing approach is adopted:

1. Track interpolation smoothing: For tracks with short-term breaks (interval ≤5 frames), linear interpolation is performed based on a kinematic model (constant acceleration) to complete the tracks;
2. Manual verification and correction: The annotation team uses the CVAT tool to manually merge tracks with abnormal ID switches, ensuring ID consistency in the final dataset.

Following post-processing optimization, the dataset’s trajectory count increased to 665(comprising 350 avian and 315 unmanned aerial vehicle trajectories), with a mean duration of 48 frames. The fragmentation rate was reduced to 4.3 %, thus meeting the requirements for spatio-temporal feature modeling. This comparative experiment not only provides methodological support for dataset construction but also offers empirical reference for algorithm selection in low-altitude Multiple Object Tracking (MOT) scenarios.

(4) Baseline Benchmark Experiments to Demonstrate LAT-BirdDrone Utility

We conducted a comprehensive comparison of several mainstream models, including Convolutional Neural Networks (CNN) [23,24], Transformer models, improved Transformer models (iTransformer), Long Short-Term Memory networks (LSTM) [25,26], Bidirectional Long Short-Term Memory networks (BiLSTM) [25], and Gated Recurrent Units (GRU) [25,26]. These models were applied to classification tasks involving birds [[27], [28], [29]] and drones. Initially, each of these models was trained independently and their performance was evaluated on the datasets. Subsequently, we tested existing hybrid architectures (e.g., Transformer+LSTM, CNN+BiLSTM) as baseline experiments, aiming to demonstrate the utility of LAT-BirdDrone in supporting multi-architecture model evaluation [30,31]. The hybrid models included combinations such as Transformer+LSTM, CNN+BiLSTM, iTransformer+CNN, Transformer+LSTM+CNN, and iTransformer+CNN+GRU. The performance results of these models are detailed in Table 4. Furthermore, we compiled statistics on the correctly and incorrectly classified trajectories for both birds and UAVs, as presented in Table 3. This comprehensive analysis provided insights into the effectiveness of different model architectures and their combinations for the specified classification tasks.

**Table 2:** Comparative experimental results of ByteTrack algorithm and BoT-SORT algorithm.

Index	ByteTrack	BoT-SORT
Track Count	184	162
ID Switch	27	15
Frag.Rate	18.2	12.7
MOTA	72.4	75.8

Note: Table 2 demonstrates a performance comparison between the ByteTrack and BoT-SORT algorithms under the same detection input, focusing on metrics including track count, ID Switch, frag.rate, and MOTA.

**Table 3:** Baseline benchmark of trajectory classification results (birds vs. drones) on LAT-BirdDrone.

Type	Model	Correct Trajectories	Incorrect Trajectories
Bird	CNN Transformer LSTM iTransformer LSTMTrans CNNBiLSTM CNNiTrans CNNLSTMTrans CNNGRUiTrans	312 299 285 320 293 **320** **330** **304** **300**	45 35 49 37 41 **37** **27** **30** **34**
Drone	CNN Transformer LSTM iTransformer LSTMTrans CNNBiLSTM CNNiTrans CNNLSTMTrans CNNGRUiTrans	294 275 261 257 284 **287** **283** **307** **309**	15 57 71 52 48 **22** **26** **25** **23**

**Table 4:** Baseline benchmark of trajectory classification performance metrics on LAT-BirdDrone.

Type	Dataset	Model	Accuracy	Precision	Recal	F1	ROC-AUC
Bird Drone	Val Test	CNN Transformer LSTM iTransformer LSTMTrans CNNBiLSTM CNNiTrans CNNLSTMTrans CNNGRUiTrans CNN Transformer LSTM iTransformer LSTMTrans CNNBiLSTM CNNiTrans CNNLSTMTrans CNNGRUiTrans	90.66 82.83 81.33 85.54 84.94 **90.96** **90.06** **89.76** **89.76** 90.99 86.19 81.98 86.64 86.64 **91.14** **92.04** **91.74** **91.44**	92.90 82.18 80.57 84.78 84.00 **92.44** **89.07** **93.55** **93.55** 95.41 83.99 80.06 86.02 85.92 **93.57** **92.70** **92.40** **92.88**	89.20 84.62 83.43 88.64 86.98 **90.34** **92.61** **85.80** **85.80** 87.39 89.52 85.33 89.64 87.72 **89.64** **92.44** **91.02** **89.82**	91.10 83.38 81.98 86.67 85.47 **91.38** **90.81** **89.51** **89.51** 91.23 86.67 82.61 87.79 86.81 **91.56** **92.57** **91.70** **91.32**	97.10 91.93 89.85 92.41 91.77 **97.35** **97.10** **95.57** **95.39** 98.36 93.60 91.30 92.86 93.79 **97.97** **98.21** **97.75** **97.81**
	Val Test	CNN Transformer LSTM iTransformer LSTMTrans CNNBiLSTM CNNiTrans CNNLSTMTrans CNNGRUiTrans CNN Transformer LSTM iTransformer LSTMTrans CNNBiLSTM CNNiTrans CNNLSTMTrans CNNGRUiTrans	90.66 82.83 81.33 85.54 84.94 **90.96** **90.06** **89.76** **89.76** 90.66 86.19 81.98 86.64 86.64 **91.14** **92.04** **91.74** **91.44**	88.34 83.54 82.17 86.49 85.99 **89.38** **91.28** **86.44** **86.44** 86.73 88.71 84.19 87.41 87.38 **88.58** **91.29** **91.10** **90.09**	92.31 80.98 79.14 82.05 82.82 **91.67** **87.18** **93.87** **93.87** 95.15 82.83 78.61 83.17 85.54 **92.88** **91.59** **92.47** **93.07**	90.28 82.24 80.63 84.21 84.38 **90.51** **89.18** **90.00** **90.00** 90.74 85.67 81.31 85.24 86.45 **90.68** **91.44** **91.78** **91.56**	97.10 91.93 89.85 92.41 91.77 **97.35** **97.91** **95.57** **95.39** 98.36 93.60 91.30 92.86 93.79 **97.97** **98.21** **97.75** **97.81**

*Note*: Val and Test sets are split at the video level (stratified sampling, Train: Val:Test = 60 %:20 %:20 %) to avoid data leakage; see “Data Splitting Strategy” for details. Trajectory statistics (e.g., count by species/model) are provided in Table 1.

Firstly, in terms of multi-architecture model support, the baseline experiments encompassed single models (such as CNN, Transformer, LSTM) and hybrid architectures (such as CNN+BiLSTM, Transformer+LSTM). All models were able to produce effective results based on the LAT-BirdDrone dataset, demonstrating that the dataset can serve as a unified benchmark for “low-altitude micro-target trajectory classification,” accommodating evaluation needs for different types of models. Secondly, regarding high annotation quality and reliability, after optimization with ByteTrack, the dataset's trajectory fragmentation rate was reduced to 4.3 %. The 665 complete trajectories enabled models (such as CNNiTrans) to achieve an accuracy of 92.04 % and a ROC-AUC of 98.21 % on the test set, indicating that the dataset's trajectory annotations have high continuity and class separability, providing a reliable basis for model training and testing. Finally, with respect to complex scene coverage and practicality, the dataset includes six types of backgrounds (urban, farmland, lake), special lighting conditions (dawn/dusk), and adverse weather (rain, snow, sandstorm). The baseline results showed that hybrid models performed consistently well on samples with complex backgrounds, demonstrating that the dataset can simulate real low-altitude monitoring environments, thus supporting subsequent applications of models in real-world scenarios.

(5) Trajectory Classification Visualization

**Fig. 16:**
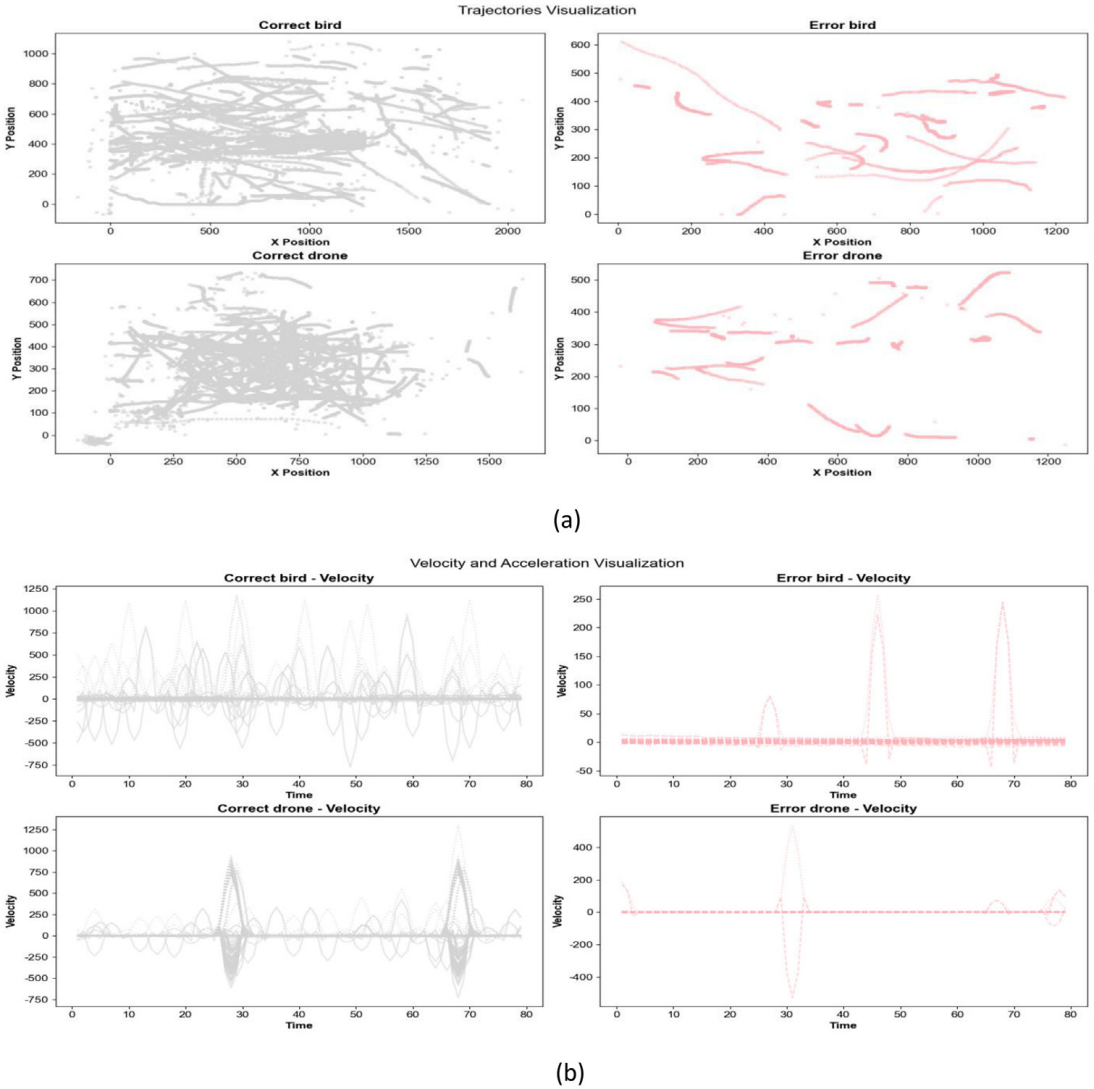
Transformer+LSTM+CNN bird and drone trajectory density maps.

We used the Transformer+LSTM+CNN hybrid model, which demonstrated high classification accuracy, to classify birds and drones, and obtained relevant images of correct and incorrect trajectories. Trajectory classification visualization and velocity distributionhistograms aid in analyzing classification errors and optimizing models. Trajectory classificationvisualization demonstrates spatial differences between correctly and incorrectly classified samples, typically represented on a 2D plane. It reveals motion patterns misclassi- fied by the model, such as rapidly turning birds misidentified as drones. This visualization guides feature engineering, especially for easily misclassified patterns (Fig. 16a). Speed-time curves provide a temporal analysis of motion patterns by visualizing speed variations over time, offering critical insights into classification performance across different object categories. This analytical approach reveals characteristic movement signatures: drones typically exhibit intermittent high-speed bursts with rapid acceleration phases, while birds demonstrate more stable speed profiles with periodic flapping-induced fluctuations (Fig. 16b).

**Fig. 17:**
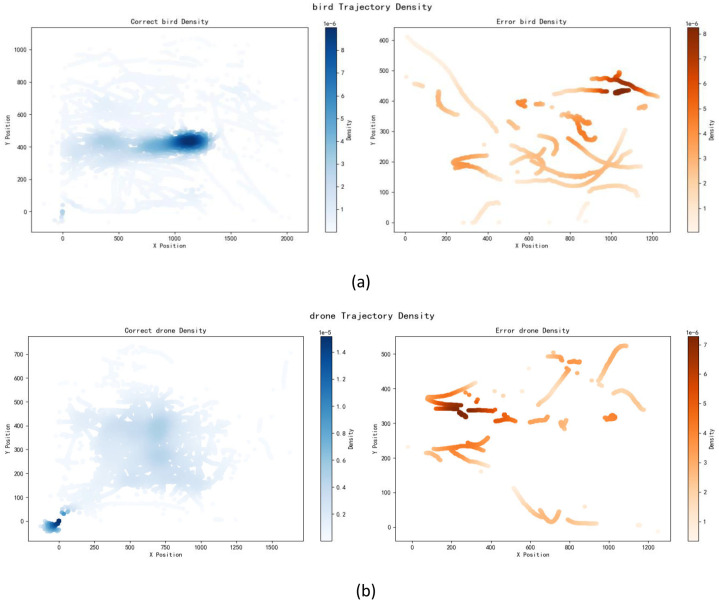
Transformer+LSTM+CNN bird and drone trajectory density maps.

Trajectory density maps use color gradients to show sample distribution density, revealing hotspots with high misclassification rates. These provide reference for model optimization, particularly significant for improvements in difficult-to-classify areas (Fig. 17).

**Fig. 18:**
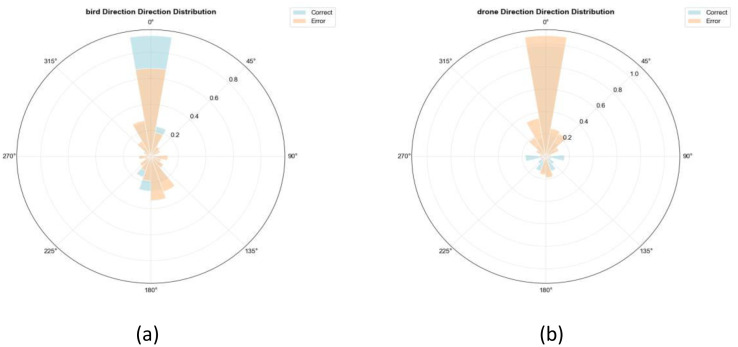
Direction distribution diagram (polar coordinates).

This polar histogram (Fig. 18) visually depicts the directional concentration trends in the trajectories of birds and drones. By mapping angular distributions, it reveals the dominant movement directions and potential orientation preferences inherent in their flight patterns.

**Fig. 19:**
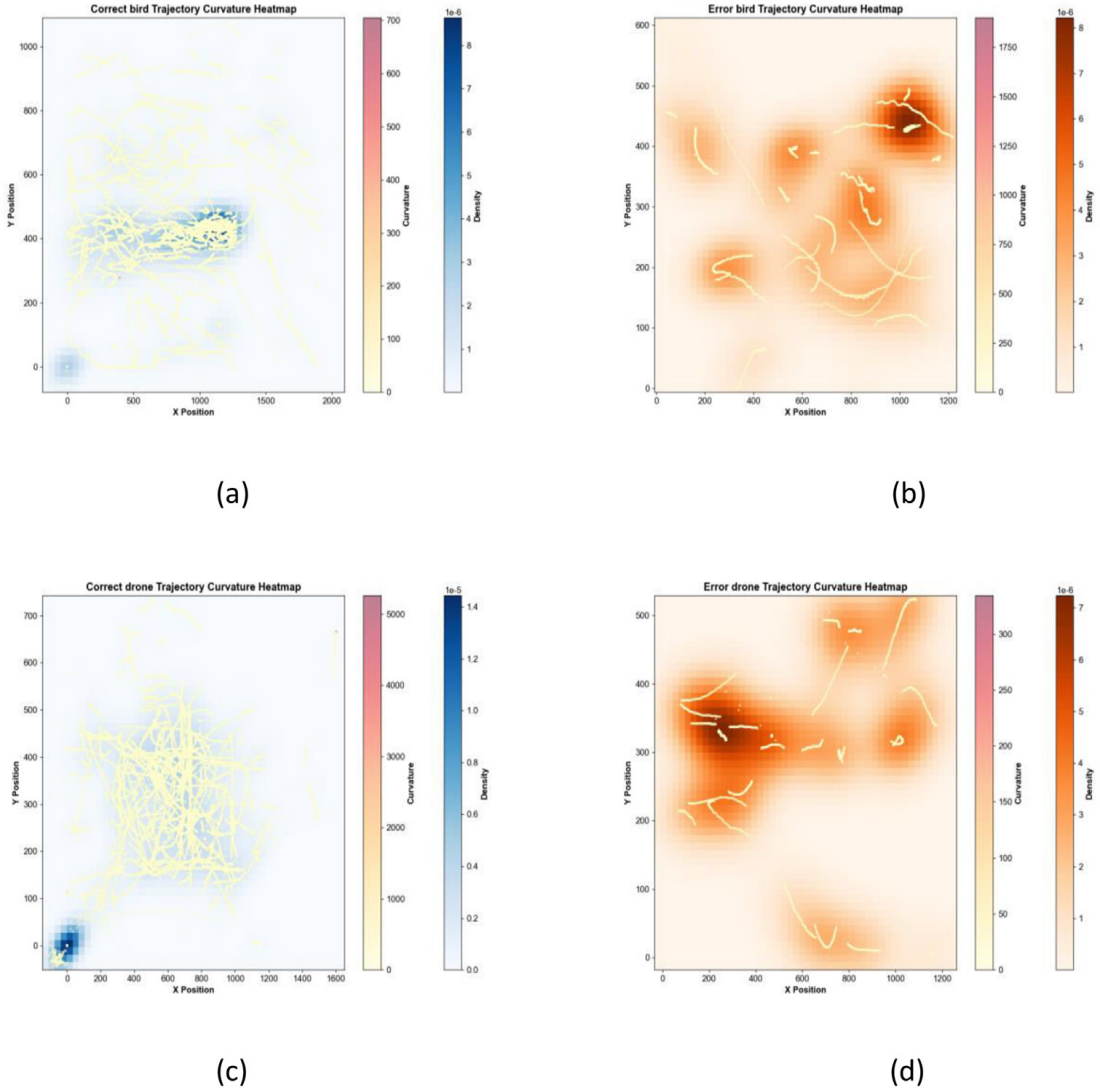
Curvature heatmap.

This two-dimensional heatmap (Fig. 19) correlates spatial positions with curvature values to identify regions of high curvature—such as sharp turns or hovering behavior—in the trajectories of birds and drones. Intense color gradients highlight areas where rapid changes in direction occur, providing insights into maneuvering strategies and environmental interactions.

**Fig. 20:**
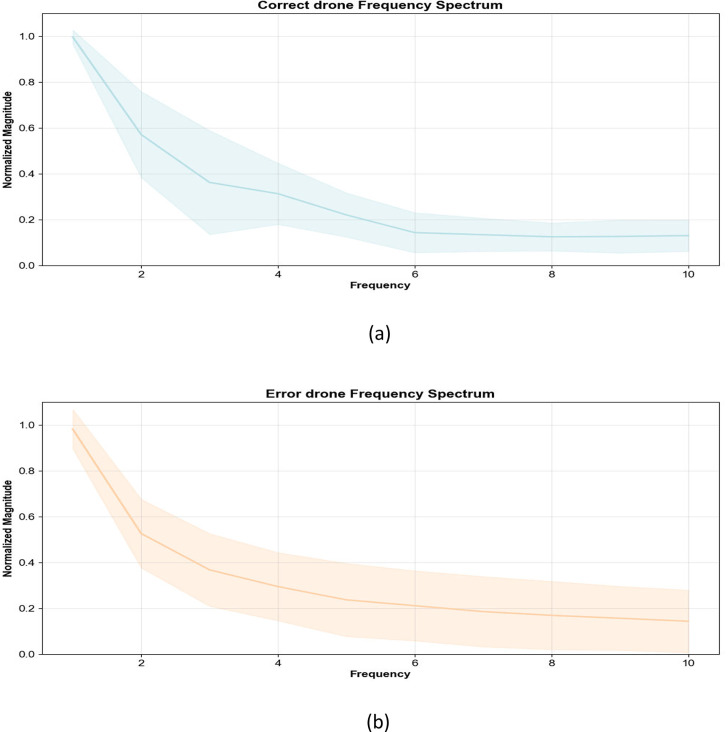
Fourier spectrum analysis.

The frequency spectrum diagram (Fig. 20) identifies periodic components in both normal and abnormal trajectories of drones. Frequency peaks correspond to specific cycle engths, allowing the detection of rhythmic patterns, mechanical vibrations (e.g., propeller- induced oscillations), or systematic errors that may indicate malfunctions or deviations from standard flight paths.

## Dataset limitations

This study exhibits several limitations that warrant in-depth discussion, as these constraints not only reflect potential gaps in the current work but also provide actionable directions for future dataset optimization and model refinement. Firstly, the LAT-BirdDrone dataset presents a notable class imbalance due to the unequal ratio of drone-to-bird videos, with 15 drone-only videos and 50 bird-only videos resulting in a ratio of 1:3.3; this imbalance directly impacts the training and validation of trajectory classification models. During training, the model tends to prioritize learning features of the majority class (birds) to minimize overall loss, leading to biased generalization—while it may achieve high accuracy in bird trajectory classification, the misclassification rate for the minority class (drones) increases significantly. For instance, experimental results of single models (Table 3) show that the CNN model misclassified only 15 drone trajectories but 45 bird trajectories, partially reflecting the model’s over-adaptation to bird features. In validation, the imbalanced sample distribution may overestimate the model’s overall performance: since bird samples account for a larger proportion of the validation set, metrics such as accuracy and F1-score are skewed toward bird classification results, failing to truly reflect the model’s ability to recognize drone trajectories—a key target for low-altitude security applications. Future solutions to this issue include augmenting drone video data via techniques like frame interpolation or synthetic trajectory generation, adopting class-balanced loss functions (e.g., focal loss) to adjust the weight of minority-class samples during training, and collecting additional drone flight videos to balance the sample ratio.

Secondly, the dataset’s limited diversity in target types—covering only 4 drone models (DJI Air, DJI Inspire, Mavic Pro, DJI Mini) and 4 bird species (crow, sparrow, goose, magpie)—restricts its generalization ability and that of the trained models to real-world low-altitude targets. For drones, the selected models are all consumer-grade mainstream products from DJI, excluding other types of low-altitude small targets such as industrial micro-drones (e.g., Parrot Anafi) or custom-made mini-drones; these unincluded drones may exhibit distinct motion patterns (e.g., slower hovering speeds for industrial drones) or appearance features (e.g., different numbers of propellers), which could reduce the model’s classification accuracy when encountering such targets. For birds, the 4 selected species were chosen for their visual similarity to drones but do not cover small birds (e.g., swallows) or large birds of prey (e.g., eagles) common in low-altitude environments; these excluded birds have more diverse flight dynamics (e.g., gliding in eagles vs. flapping in sparrows) that the current dataset fails to capture, limiting the model’s adaptability to varied avian motion patterns. Subsequent dataset updates should expand the range of target types to include non-DJI drone models and additional bird species, thereby enhancing the dataset’s representativeness for real low-altitude monitoring scenarios.

Thirdly, the dataset’s mean trajectory length of 48 frames (equivalent to ∼1.92 s at 25 fps) poses challenges for temporal modeling of extremely short or extremely long trajectories. For extremely short trajectories (e.g., <10 frames, ∼0.4 s), the limited number of frames fails to capture sufficient spatiotemporal features (e.g., short-term acceleration or direction changes of targets), leading to high fragmentation rates even after post-processing (current fragmentation rate: 4.3 %) and increasing the model’s difficulty in distinguishing between transient drone movements (e.g., sudden takeoffs) and bird movements (e.g., quick escapes). For extremely long trajectories (e.g., >100 frames, ∼4 s), the current mean length does not cover long-duration motion patterns (e.g., continuous cruising of drones or long-distance gliding of birds); models relying on temporal dependencies (e.g., LSTM, Transformer) may suffer from long-sequence information degradation—for example, the gradient vanishing issue in LSTM could weaken its ability to capture long-term motion trends of targets. Future improvements to address this limitation include collecting trajectory data of varying durations to cover both short (<10 frames) and long (>100 frames) sequences, optimizing trajectory segmentation strategies (e.g., sliding window methods) to adapt to long sequences, and adopting models specialized in long-sequence modeling (e.g., Transformer-XL) to complement the current temporal modeling framework.

## Conclusions

Although the LAT-BirdDrone dataset provides an important resource for trajectory classification of low-altitude flying small targets, we recognize that the dataset still has certain limitations in terms of scale. The dataset used in this study comprises 65 videos (15 of drones and 50 of birds) and 665 trajectories, with an average of 48 frames per trajectory. The relatively small number of videos and trajectories may limit the ability to achieve high-precision classification.To overcome these limitations, we plan to expand the LAT-BirdDrone dataset by increasing the scale and diversity of data collection, involving more types of drones and birds. Additionally, we aim to include more diverse target types, encompassing low-altitude flying objects of different sizes, shapes, and motion characteristics. We will also gather data from more complex scenes, such as adverse weather conditions and intricate background environments, and extend the trajectory lengths beyond the current average of 48 frames to support more complex temporal modeling. Moreover, we will consider adding multi-perspective data to provide more comprehensive information about target movements.

These efforts will help to better validate and improve trajectory classification models and advance the field of low-altitude small target monitoring. Through collaborative efforts and the inclusion of diverse data sources, we believe that we can construct a more comprehensive and practically valuable dataset.

## Limitations

None

## Ethics Statement

We hereby confirm that the authors have read and follow the ethical requirements for publication in Data in Brief. Additionally, we confirm that the current work does not involve human subjects, animal experiments, or any data collected from social media platforms.

## CRediT Author Statement

**Ming Ke:** Conceptualization, Methodology, Software; **Xin Kang**: Data curation, Writing, Original draft preparation; **Zixuan Zhang**: Visualization, Investigation; **Lubing Wang:** Supervision; **Gang Wang**: Software, Validation.

## Data Availability

ScienceDBLAT-BirdDrone Dataset (Original data).
